# Understanding the role of mesenchymal stem cells in urinary bladder regeneration—a preclinical study on a porcine model

**DOI:** 10.1186/s13287-018-1070-3

**Published:** 2018-11-28

**Authors:** Marta Pokrywczynska, Arkadiusz Jundzill, Marta Rasmus, Jan Adamowicz, Daria Balcerczyk, Monika Buhl, Karolina Warda, Lukasz Buchholz, Maciej Gagat, Dariusz Grzanka, Tomasz Drewa

**Affiliations:** 1 Department of Regenerative Medicine, Cell and Tissue Bank, Chair of Urology, Nicolaus Copernicus University in Torun, Ludwik Rydygier Medical College in Bydgoszcz, Marii Sklodowskiej Curie 9 Street, 85-094 Bydgoszcz, Poland; 2 Department of Embryology and Histology, Nicolaus Copernicus University in Torun, Ludwik Rydygier Medical College in Bydgoszcz, 85-092 Bydgoszcz, Poland; 3 Department of Clinical Pathomorphology, Nicolaus Copernicus University in Torun, Ludwik Rydygier Medical College in Bydgoszcz, 85-094 Bydgoszcz, Poland

**Keywords:** Urinary bladder, Regeneration, Tissue engineering, Large animal model, Stem cells

## Abstract

**Background:**

The tissue engineering of urinary bladder advances rapidly reflecting clinical need for a new kind of therapeutic solution for patients requiring urinary bladder replacement. Majority of the bladder augmentation studies have been performed in small rodent or rabbit models. Insufficient number of studies examining regenerative capacity of tissue-engineered graft in urinary bladder augmentation in a large animal model does not allow for successful translation of this technology to the clinical setting. The aim of this study was to evaluate the role of adipose-derived stem cells (ADSCs) in regeneration of clinically significant urinary bladder wall defect in a large animal model.

**Methods:**

ADSCs isolated from a superficial abdominal Camper’s fascia were labeled with PKH-26 tracking dye and subsequently seeded into bladder acellular matrix (BAM) grafts. Pigs underwent hemicystectomy followed by augmentation cystoplasty with BAM only (*n* = 10) or BAM seeded with autologous ADSCs (*n* = 10). Reconstructed bladders were subjected to macroscopic, histological, immunofluoresence, molecular, and radiological evaluations at 3 months post-augmentation.

**Results:**

Sixteen animals (*n* = 8 for each group) survived the 3-month follow-up without serious complications. Tissue-engineered bladder function was normal without any signs of post-voiding urine residual in bladders and in the upper urinary tracts. ADSCs enhanced regeneration of tissue-engineered urinary bladder but the process was incomplete in the central graft region. Only a small percentage of implanted ADSCs survived and differentiated into smooth muscle and endothelial cells.

**Conclusions:**

The data demonstrate that ADSCs support regeneration of large defects of the urinary bladder wall but the process is incomplete in the central graft region. Stem cells enhance urinary bladder regeneration indirectly through paracrine effect.

**Electronic supplementary material:**

The online version of this article (10.1186/s13287-018-1070-3) contains supplementary material, which is available to authorized users.

## Background

Bladder augmentation has been established as the main surgical option for the treatment of high-pressure, small-volume, noncompliant bladder [1]. In 1899, Mikulicz introduced a possibility to utilize a small intestine for replacement of the bladder wall [2]. This concept gained popularity and is a leading strategy in reconstructive urology to date despite numerous complications associated with the use of an intestinal tissue including metabolic disturbance, neurologic disorders, bone demineralization, and potential malignant transformation. In 2005, Atala et al. demonstrated that tissue engineering might offer a feasible alternative to bladder augmentation based on intestinal segment [3]. Unfortunately, the more recent study by Joseph et al. was not able confirm these results [4]. Majority of the bladder augmentation preclinical studies have been performed in small rodent or rabbit models. Insufficient number of studies examining regenerative capacity of tissue-engineered graft in urinary bladder augmentation in a large animal model does not allow for successful translation of this technology to the clinical setting.

Two major approaches of urinary bladder regeneration by tissue engineering include the use of cellular or acellular scaffolds. The acellular scaffold approach involves different biomaterials that stimulate in vivo spontaneous regeneration by serving as a solid support for the ingrowth of urothelial and smooth muscle patient’s native cells. The cellular scaffold approach involves autologous patient’s cells expanded in vitro and seeded into biomaterials to form neo-bladder tissue in vitro and subsequently fully regenerate bladder tissues in vivo [5]. The major challenge of tissue engineering approach is to generate graft suitable in terms of biomechanics and biocompatibility for bladder wall substitution. Throughout the last decades, none of introduced biomaterials fulfilled these criteria [6]. Matrices derived from natural tissues like small intestinal submucosa (SIS) or bladder acellular matrix (BAM) seem to be an ideal option. Bladder acellular matrix is characterized by excellent mechanical (elasticity, strength) and biological properties (low immunogenenicity, biodegradable, and bioresorbable). It consists of a complex of functional and structural proteins including collagen (types I and III), elastin, fibronectin, glycosaminoglycans (GAG), and proteoglycans in proportions compared with normal bladder tissue [7].

Cells enhance regeneration of tissue-engineered urinary bladder preventing fibrosis and scar formation [8–10]. Both differentiated urothelial and smooth muscle cells and undifferentiated stem cells were used for urinary bladder regeneration [3, 4, 8–11]. In our previous study, we found that source of mesenchymal stem cells (bone morrow vs adipose tissue) had no impact on regeneration of tissue-engineered urinary bladder. However, regeneration outcome is strictly dependent on the number of cells used for the reconstruction [12].

Despite numerous studies on urinary bladder reconstruction using stem cells, the cellular mechanisms underlying regeneration remain poorly understood. It is unknown how undifferentiated stem cells enhance urinary bladder regeneration. One possible mechanism includes direct differentiation of implanted stem cells into desired cell types under signals from surrounding microenvironment of bladder tissue. Stem cells can also enhance urinary bladder regeneration indirectly; they can stay undifferentiated and release growth factors or dye under toxic influence of urine and act as a feeder layer rich in trophic factors that trigger migration of native cells from surrounding tissues. Therefore, the purpose of this study was to evaluate the role of adipose-derived stem cells (ADSCs) in regeneration of clinically significant urinary bladder wall defect in a large animal model. The experiment workflow is presented in the Additional file 1: Figure S1.

## Materials and methods

### Ethics statement

The study was carried out in strict accordance with recommendations from the Guide for the Care and Use of Laboratory Animals of the National Institutes of Health. The research protocol was approved by the Nicolaus Copernicus University Ethics Committee (no. 25/2014).

### Adipose-derived stem cell isolation, culture, and characterization

Adipose tissue was harvested from a superficial abdominal Camper’s fascia in 10 anesthetized female domestic pigs. ADSC isolation and establishing of primary cell culture were done as previously described. To confirm ADSC immunophenotype, cells from the third final passage were incubated with CD11b, CD29, CD31, CD44, CD45, and CD90 (Abcam, UK; BD Biosciences, USA; GeneTex, USA) monoclonal antibodies conjugated with fluorescein isothiocyanate (FITC) or phycoerythrin (PE) in accordance with the manufacturer’s instructions. FITC- or PE-conjugated IgG1κ were used as the isotype controls (Abcam, UK; BD Biosciences, USA). Data were collected using BD FACSCanto II and analyzed with BD FACSDiva™ Software (BD Biosciences, USA). Evaluation of ADSCs’ multipotency was done by testing their adipogenic, chondrogenic, and osteogenic differentiation potential. ADSCs were cultured in selective, commercially available differentiation media in accordance with the manufacturer’s instructions including Mesenchymal Adipogenesis Kit (Merck Millipore, USA), StemPro™ Osteogenesis Differentiation Kit (Gibco, Thermo Fisher Scientific, USA), and StemPro™ Chondrogenesis Differentiation Kit (Gibco, Thermo Fisher Scientific, USA). Adipogenesis, chondrogenesis, and osteogenesis were confirmed by Oil Red O (Merck Millipore, USA), Alcian Blue (Sigma-Aldrich, Germany), and Alizarin Red (Merk Millipore, USA) stainings, respectively.

### Bladder acellular matrix preparation

Urinary bladders were harvested from 20 adult female pigs (~ 100 kg body weight, 6 months old) at a local abattoir. The bladders were decellularized in a housemaid perfusion system using 0.5% Triton X-100 (Sigma-Aldrich, Germany) and 26.5 mM ammonium hydroxide (Honeywell, USA) solution within 14 days. Decellularization effectiveness was tested by scanning electron microscopy (SEM) (Auriga 60, Zeiss, Germany).

### Bladder acellular matrix cytotoxicity

**Fig. 1:**
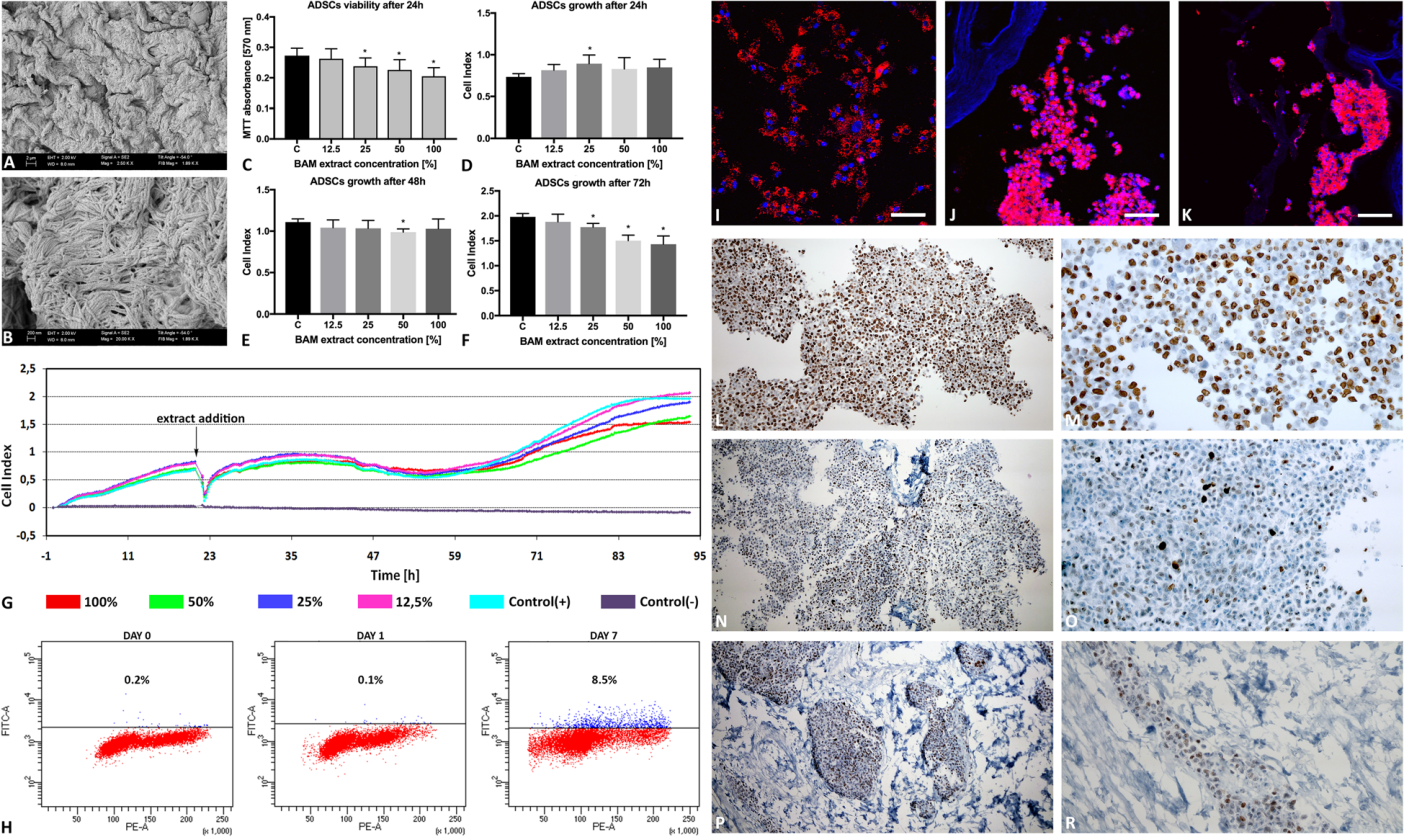
Characterization of tissue-engineered graft for urinary bladder augmentation. **a**, **b** Bladder acellular matrix microstructure. Scanning electron microscopy (bar 2 μm, 200 nm). **c–g** Analysis of bladder acellular matrix (BAM) extract cytotoxicity. **c** Viability of ADSCs after 24 h exposition to 12.5, 25, 50, and 100% BAM extracts, MTT assay. **d–g** Growth of ADSCs in real time after 24, 48, and 72 h exposition to 12.5, 25, 50, and 100% BAM extracts, X-Celligence, *p < 0.05. **h** Flow cytometric quantification of PKH-26-labeled ADSC apoptosis before and 1 day and 7 days after the seeding on BAM, respectively. **i**–**k** PKH-26 fluorescently labeled ADSCs (red) before and 1 day and 7 days after the seeding on BAM, respectively. Cell nuclei counterstained with DAPI (blue), scale bars = 100 μm. **l–r** Immunohistochemical staining of PKH-26-labeled Ki-67 in ADSCs seeded on BAM in short 1-day (**l**, **m**) and long 7-day (**n**–**r**) culture, an objective magnification × 4 and × 10. ADSCs forming multilayer on the surface of the BAM (**l–o**) and migrating through BAM are observed (**p**, **r**)

The indirect cytotoxicity assay, compliant with ISO 10993, was performed. BAM extracts were prepared by incubation of scaffold fragments in growth medium (1:1 weight ratio, 1 ml = 1 mg). Four extract concentrations (100%, 50%, 25%, and 12.5%) were used to observe a dose-response relationship (Fig. 1). ADSCs were exposed to freshly prepared BAM extracts or growth medium (control) for 24 h (MTT) or 72 h (X-Celligence system Real-Time Cell Analyzer, RTCA, ACEA Biosciences Inc., USA).

### Graft preparation: adipose-derived stem cell labeling with PKH-26 tracking dye

ADSCs labeled with PKH26 (Sigma-Aldrich, Germany) were seeded on BAM scaffolds (25 cm^2)^ at a density of 15 × 10^6^ cells/cm^2^ and cultured for 7 days. ADSC morphology and their spatial distribution was assessed by SEM. PKH-26-labeled ADSCs growth on BAM was analyzed in a confocal laser scanning microscope. For this purpose, ADSC-seeded BAMs were frozen on dry ice and sectioned at 10-μm thickness with the cryostat. The sections were stained with 4′,6-diamidino-2′-phenylindole (DAPI) dihydrochloride (Sigma-Aldrich), mounted and observed under a confocal laser scanning microscope (Nikon, Japan).

### Graft characterization: proliferation and apoptosis of ADSCs seeded on BAM

Apoptosis level in PKH-26-labeled ADSCs seeded on BAM after 1- and 7-day culture was determined with APO-DIRECT™ Kit (BD Pharmingen™, USA). This method detects DNA fragmentation, which is one of the later steps in apoptosis, by terminal deoxynucleotidyltransferase dUTP nick end labeling (TUNEL). Detached cells were stained according to the manufacturer’s instructions. DNA breaks marked with FITC-dUTP were subsequently detected by flow cytometric analysis with BD FACSCanto II (BD Biosciences, USA).

Ki-67 immunohistochemical staining was used to analyze proliferation of PKH-26-labeled ADSCs seeded on BAM after 1- and 7-day culture. Grafts were fixed in 10% phosphate buffered formalin for 24 h. The paraffin blocks were cut into 3-μm-thick sections with a manual rotary microtome (Accu-Cut, Sakura Finetek, Torrance, CA, USA) and then placed on extra adhesive slides (SuperFrost Plus; Menzel-Glaser, Braunschweig, Germany). Immunohistochemical staining was performed using anti-Ki-67 (30-9) rabbit monoclonal primary antibody as a pre-diluted reagent and visualization system ultraView DAB Detection Kit (Ventana Medical Systems, Tucson, AZ, USA) on a Benchmark Ultra platform according to the manufacturer’s instructions (Ventana Medical Systems, Tucson, AZ, USA). Additionally, stained preparations were dehydrated in ethyl alcohol of increasing concentration (from 80 to 98.8%), then cleared in a series of xylenes (from I to IV) and mounted with Shandon Consul Mount (Thermo Scientific, Waltham, USA).

### Urinary bladder augmentation

**Fig. 2:**
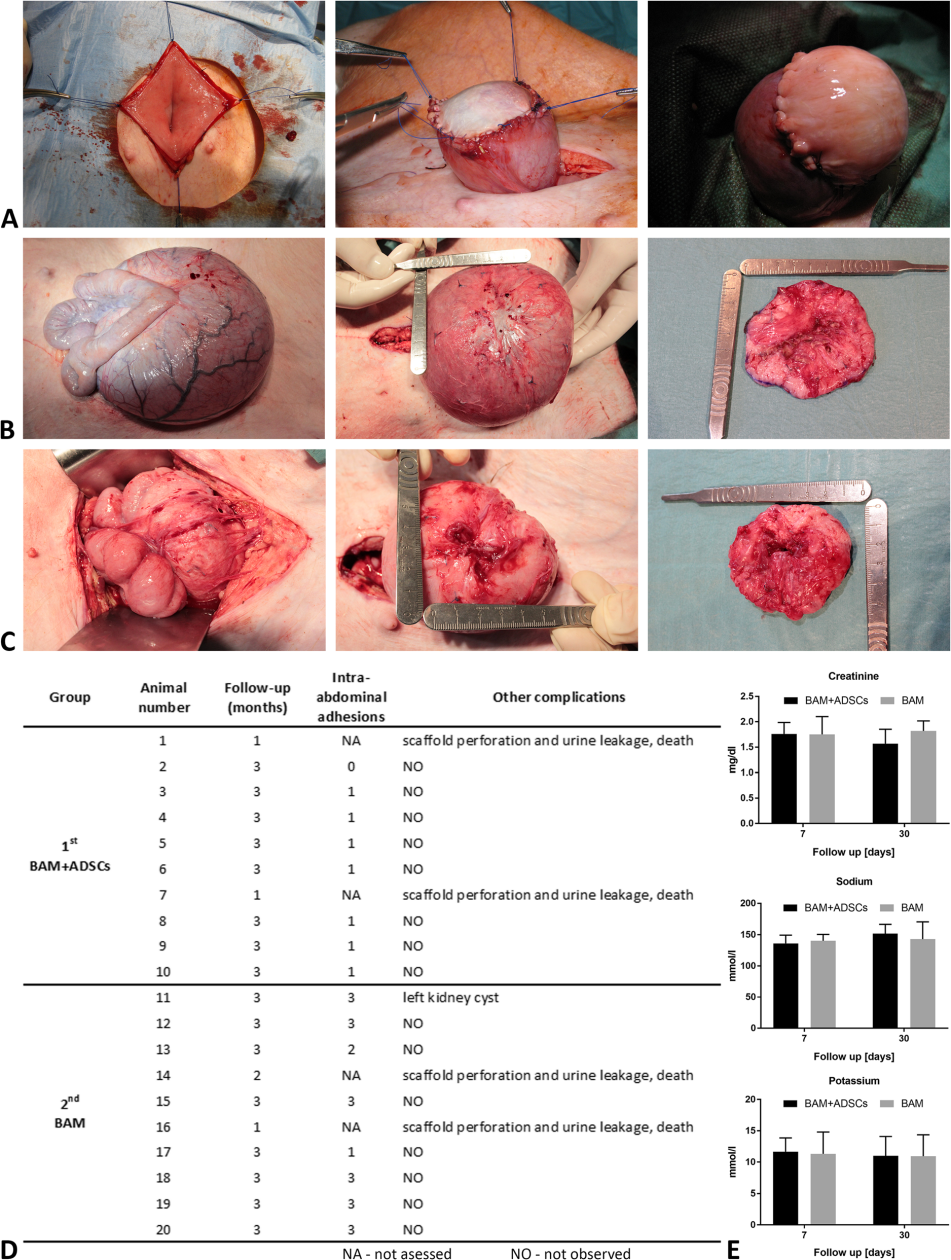
**a** Urinary bladder reconstruction: hemicystectomy, urinary bladder augmentation with stem cell seeded (**b**) and unseeded BAM (**c**): intra-abdominal adhesions, augmented bladders, and isolated reconstructed bladder walls at 3 months following the surgery. **d** Survival and complications. No stone formation, signs of infection, or hydronephrosis were observed in examined animals. Postoperative intra-abdominal adhesions were measured using the following scale: 0: lack of adhesions; 1: minimal adhesions easy to separate; 2: moderate adhesions hard to separate; 3: dense adhesions that could be separated with a sharp tool. **e** Blood levels of creatinine, sodium, and potassium were normal at 7 and 30 days postoperatively, in all animals

The surgical procedures were carried out on 20 female domestic pigs (~ 60 kg). Animals were anesthetized with ketamine (5 mg/kg)*.* The peritoneal cavity was opened by a midline abdomen incision. After urinary bladder exposition, hemicystectomy was performed (Fig. 2a). Gap’s margins were marked by eight 4-0 prolene (Ethicon, USA) sutures. Silicone 18-F Foley catheter (Rusch, Germany) was introduced into the bladder. Animals were divided into two groups: first group, *n* = 10, cellular graft—BAM seeded with ADSCs; second group, *n =* 10, acellular graft—BAM only. The urinary bladders were accordingly augmented. Two layered semicontinuous sutures were used to anastomose grafts with a native bladder wall (Fig. 2a).

### Postoperative assessments

Creatinine, electrolyte, and urine tests were done 7 and 30 days postoperatively. Computed tomography urography (CTU) was performed using a 16-row scanner (Siemens, Germany) at the end of follow-up. Based on measured bladder dimensions on axial and front cross sections, the bladder volume was estimated. The animals were sacrificed by intravenous administration of a pentobarbital overdose. Urinary bladder regeneration was evaluated in three areas: distal graft region—graft center (R1), mid-graft region (R2) and compared to native bladder wall structure (N). Urinary bladder regeneration in proximal graft region adjacent to native bladder tissue (anastomosis line) was not analyzed due to difficulties to distinguish native from regenerated bladder tissue.

### Histological staining

Urinary bladder samples were fixed in 10% phosphate buffered formalin for 24–48 h. Paraffin blocks were sectioned at the thickness of 4 μm, placed on microscope slides, and stained routinely with H&E.

### Immunofluorescence staining

The urinary bladder tissue sections were embedded in Tissue-Tek® (Sakura Finetek, USA) and frozen in liquid nitrogen. Frozen tissues were sectioned at 10 μm thickness with the cryostat. The set of following markers: pan cytokeratin (panCK), alpha smooth muscle actin (αSMA), von Willebrand Factor (vWF), S100, CD90, and CD14 antibodies (1:150; Abcam, GB; GenWay Biotech, USA; Bio-Rad, USA), were applied to analyze the quality of occurred urothelium, smooth muscles, blood vessels, nerve fiber regeneration, and macrophage infiltration. Sections were visualized by donkey anti-mouse Alexa Fluor 647 conjugate (1:300, Thermo Fisher Scientific, USA) or goat anti-rabbit Alexa Fluor 647 conjugate (1:300, Thermo Fisher Scientific, USA) secondary antibody. Cell nuclei were stained with DAPI (Sigma-Aldrich, Germany). The expression of analyzed markers and their colocalization with PKH26 dye were analyzed using a Cell Observer SD Confocal Microscope (Zeiss, Germany) according to methods described previously by Manders et al. [13]. The colocalization was measured using ImageJ: JACoP according to methods described previously by Bolte and Cordelières [14]. Manders colocalization factor (*M*) can assume the value from 0 to 1. If fluorescence signal A is completely contained in signal B, then the coefficient is 1. If there is no common part between signals A and B, then the coefficient is 0. To track cell migration to peripheral organs, the level of PKH26 dye expression was evaluated in the liver, lung, and spleen tissue sections.

### Real-time PCR

Total RNA was isolated using High Pure RNA Tissue Kit (Roche Diagnostics, Switzerland) according to the manufacturer’s protocol. Quality and quantity of isolated RNA was analyzed on NanoDrop (Thermo Scientific, USA) and Agilent 2100 BioAnalyzer (Agilent Technologies, USA) with the use of RNA 6000 Nano Kit (Agilent Technologies, USA). cDNA was synthesized from 500 ng of total RNA after genomic DNA elimination using RT^2^ First Strand Kit (Qiagen, Germany). Quantitative real-time PCR was performed with the use of RT^2^ SYBR Green Mastermix (Qiagen, Germany). RT^2^ Profiler PCR Array (Qiagen, Germany) was designed to determine expression levels of genes related to urothelium, smooth muscle, nerve, and blood vessel regeneration. The primer sequences are presented in Additional file 2: Table S1. The Roche LightCycler 480 software 1.5 (Roche Diagnostics, Switzerland) was used to perform advanced relative quantification analysis.

### Statistical analyses

The statistical differences between groups were calculated by ANOVA followed by last significant differences (LSD) or Tamhane post hoc multiple comparison tests (IBM SPSS Statistics, Predictive Solutions, Poland). Statistically significant differences were defined as having *p* < 0.05.

## Results

### Analysis of adipose-derived stem cell morphology, immunophenotype, and multipotency

ADSCs after the third passage showed spindle-shaped, fibroblast-like morphology typical for mesenchymal stem cells. Flow cytometry confirmed the ADSC immunophenotype with high expression of CD29 (87.74% ± 8.08), CD44 (93.50% ± 6.29), and CD90 (99.60% ± 0.44) surface markers and low expression of CD11b (0.23% ± 0.10), CD31 (13.23% ± 5.95), and CD45 (0.62% ± 0.40) surface markers (Additional file 3: Figure S2A). ADSCs cultured in the appropriate differentiation media could differentiate into adipogenic, chondrogenic, and osteogenic lineages (Additional file 3: Figure S2B).

### Analysis of bladder acellular matrix structure and cytotoxicity

Scanning electron microscopy has shown an intact mesh of collagen fibers with no evidence of cells or cellular debris (Fig. 1a, b). ADSCs exposed to BAM extracts retained their fibroblast-like morphology and remained attached to the bottom of the culture plates. Cell lysis was not observed. Both MTT and real-time X-Celligence assays did not show cytotoxic effect of BAM extracts on ADSCs that according to ISO 10993 standard is considered as a decrease in cell growth and viability below 70% compared to control (Fig. 1c–g). The viability of cells after 24 h exposition to 100% BAM extract decreased to 82.71% (± 6.53%) (Fig. 1c). The results of X-Celligence measurements showed initially increase in growth of cells exposed to BAM extracts (24 h) which in the following hours reached the level of control group (48 h) and subsequently decreased (72 h)(Fig. 1d–g). However, even after 72 h exposition to 100% BAM extract, the cell growth was > 70% compared to the control (Fig. 1f, g).

### Analysis of PKH-26-labeled ADSC growth on bladder acellular matrix

PKH-26-labeled ADSCs formed a multilayer on the surface (Fig. 1j, l–o) and migrated inside of BAM (Fig. 1k, p, r). Flow cytometric analysis confirmed high viability of PKH-26-labeled ADSCs before and after 1-day culture on BAM. The number of apoptotic PKH-26-labeled ADSCs increased up to ~ 10% after 7-day culture on BAM (Fig. 1h). These results are consistent with MTT and X-Celligence assays that also showed some decrease in cell viability after exposition to BAM extract. The percentage of Ki-67-positive ADSCs was 60.8 ± 3.99% in the short-term 1-day culture (Fig. 1l, m) and 9.4 ± 3.48 in the long-term 7-day culture (Fig. 1n–r). The reduction in Ki-67 expression is the cell density-dependent contact inhibition effect that can normally occur in a long-term culture.

### Survival, complications, and evaluations of reconstructed bladders

**Fig. 3:**
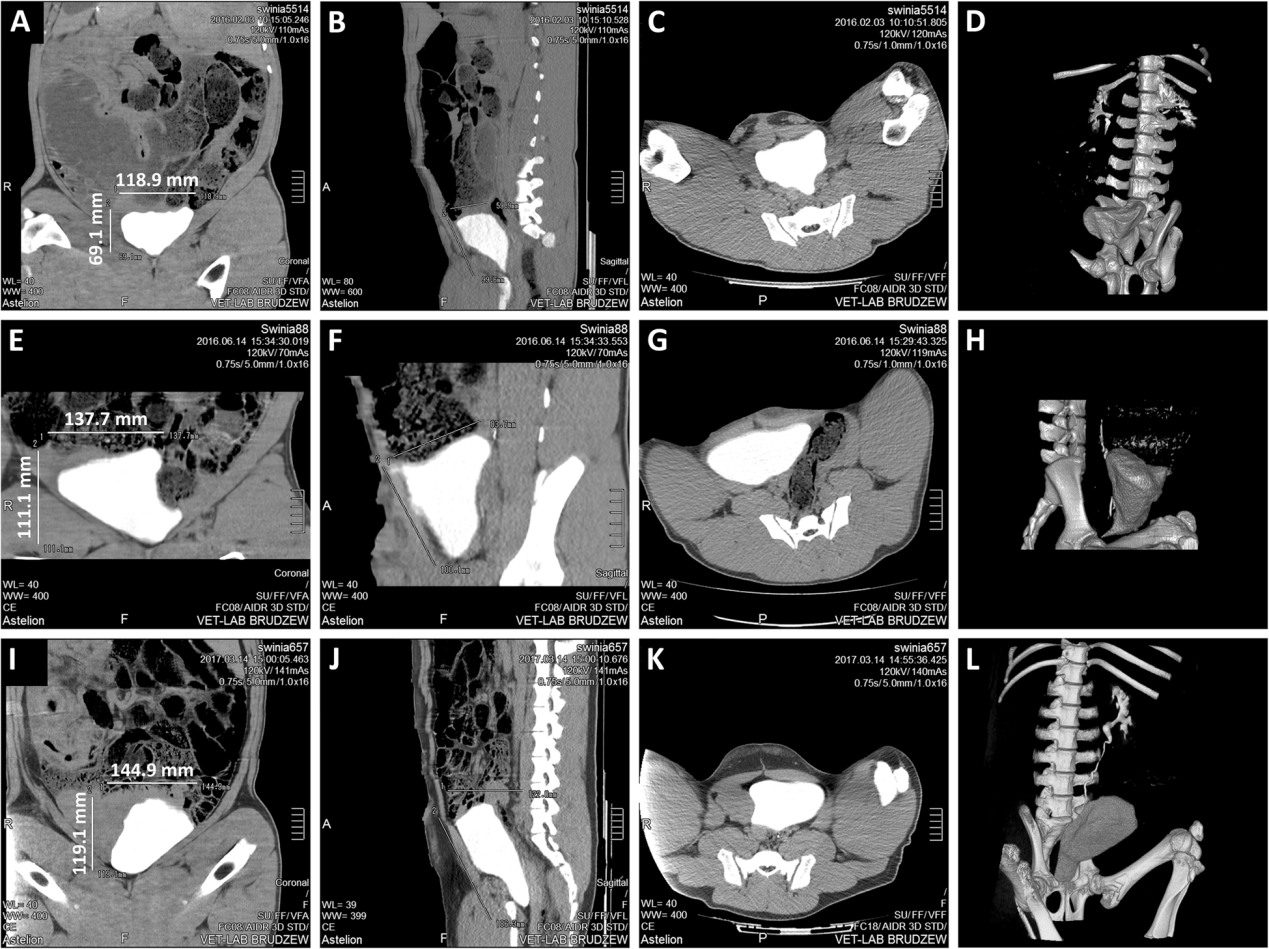
Computed tomography of porcine urinary bladders augmented with BAM only (**a–d**) and BAM seeded with ADSCs (**e–h**) at 3 months follow-up and control (**i–l**). Three-phase study with urography. Frontal, sagittal, and transverse images and 3D reconstructions show upper and lower urinary tracts. CT imaging shows the regenerated bladder wall without pathological thickening or diverticulum. CT scans of the upper urinary tract did not show pelvicalyceal system dilation or any sign of urine retention

Sixteen pigs survived the follow-up period without relevant complications (Fig. 2d). Four animals died due to scaffold perforation. The volume of bladder augmented with ADSC-seeded BAM was 1030 ± 408 ml, BAM alone—485 ± 311 ml, and control—1331 ± 539 ml. Graft’s fibrosis with consecutive shrinkage was observed in both groups (Fig. 2b, c). These processes were the most pronounced in bladders reconstructed with BAM without cells (Fig. 2c). The fibrotic reaction took place mainly in the graft center. CT imaging revealed proper morphology of augmented bladders (Fig. 3).

### Molecular analysis of urothelium, smooth muscle, nerve, and vessel regeneration

**Fig. 4:**
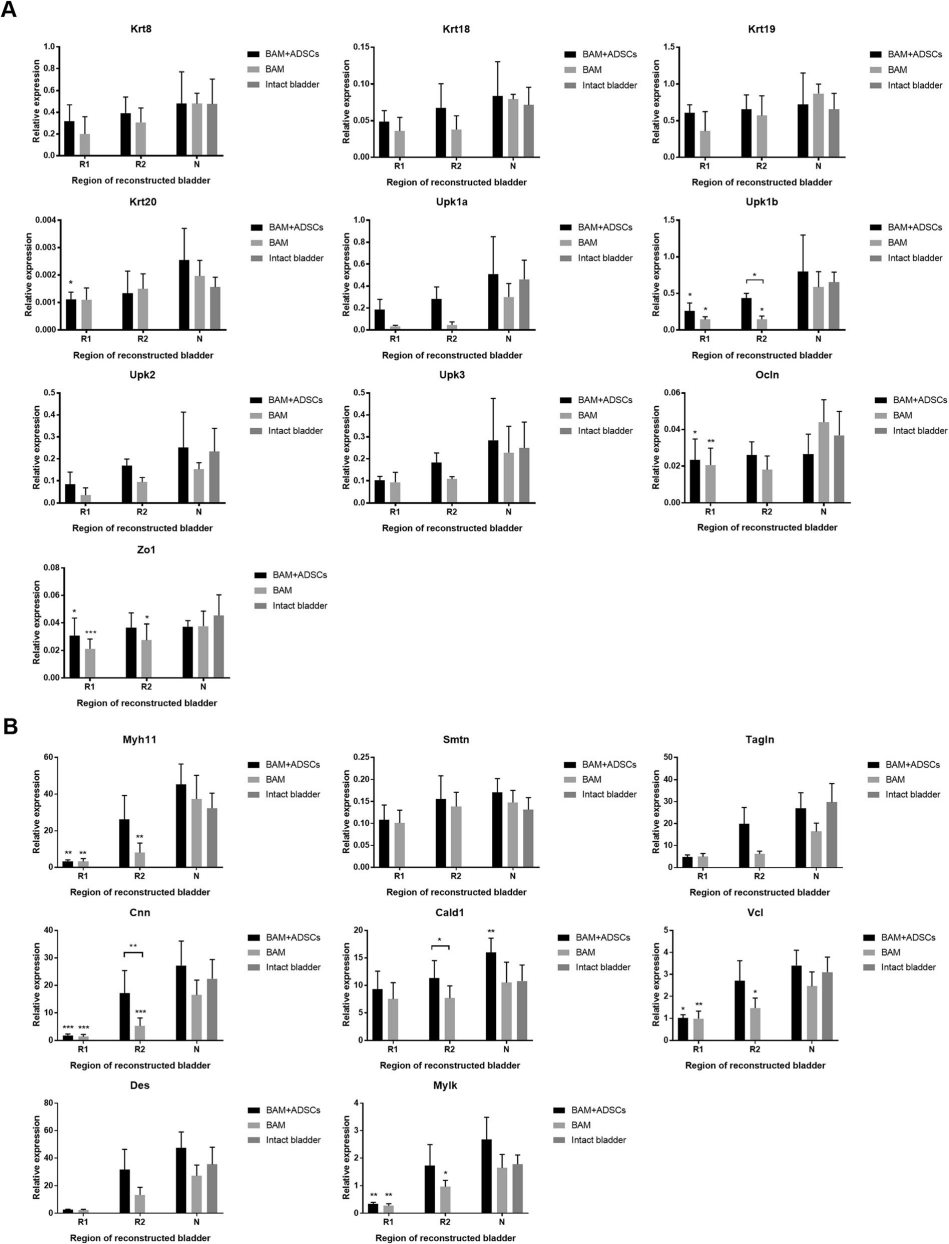
Expression of urothelial (**a**) and smooth muscle cell (**b**) markers in urinary bladders augmented with BAM seeded with ADSCs or BAM only determined by RT-PCR. R1—proximal graft region, R2—mid-graft region, N—native bladder wall in reconstructed bladders. Expression values were normalized to *Actg1* and *Hprt1*. Data are presented as mean ± SD, *p < 0.05, **p < 0.01, ***p < 0.001

Expression of smooth muscle cell markers—calponin (Cnn) and caldesmon 1 (Cald1)—was significantly higher in bladders augmented with BAM seeded with ADSCs compared to unseeded BAM (*p* < 0.01 and *p* < 0.05). Comparable to native expression of myosin heavy chain 11 (Myh11), smoothelin (Smtn), transgelin (Tagln), Cnn, Cald1, vinculin (Vcl), desmin (Des), and myosin light chain kinase (Mylk) were detected only in the mid-graft region (R2) (*p* > 0.05). In the bladders augmented with unseeded BAM expression of Myh 11, Cnn, Vcl, and Mylk in both analyzed regions, R1 and R2 had not reached the native level (Fig. 4b).

**Fig. 5:**
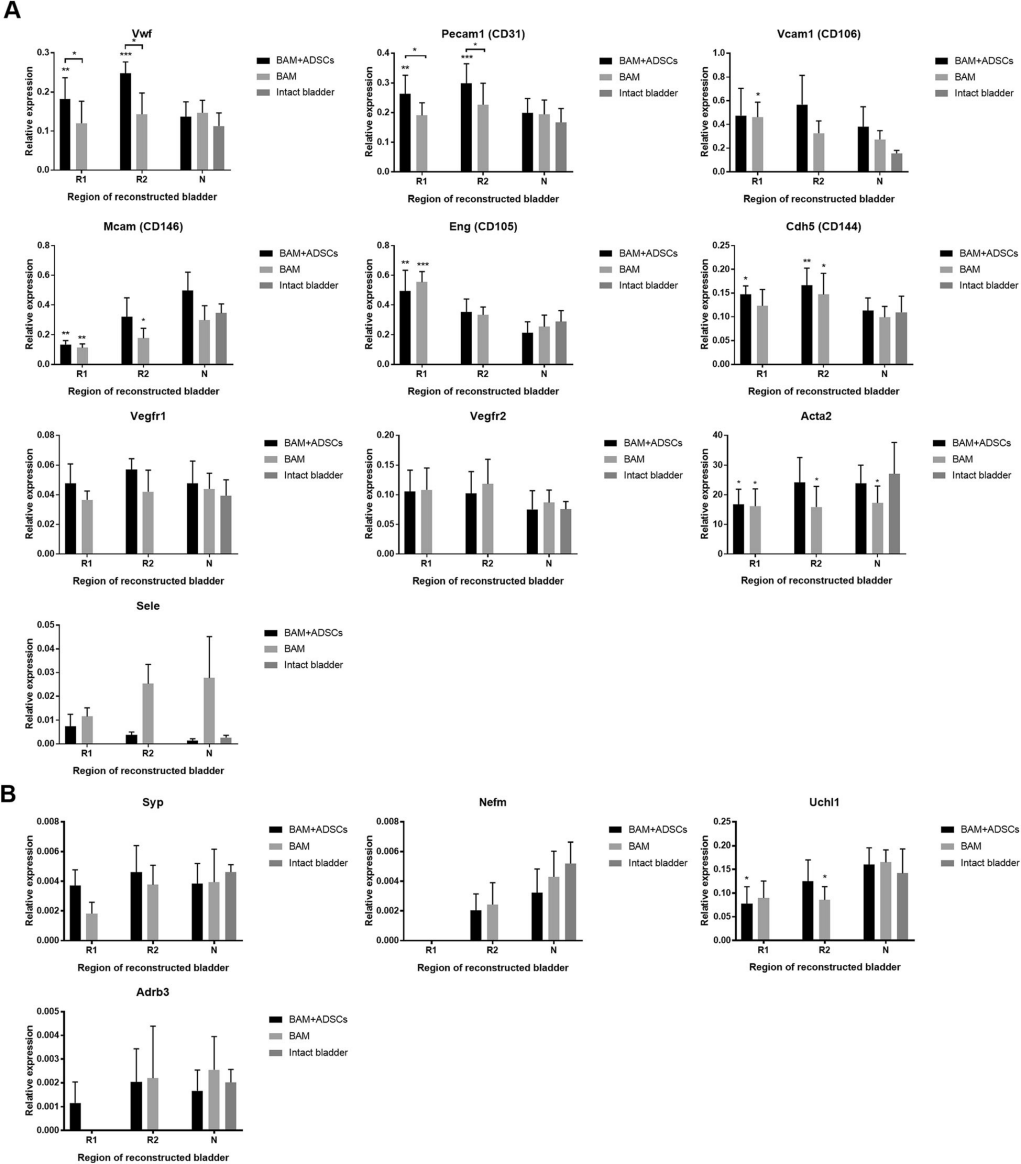
Expression of endothelial (**a**) and neuronal cell (**b**) markers in urinary bladders augmented with BAM seeded with ADSCs or BAM only determined by RT-PCR. R1—proximal graft region, R2—mid-graft region, N—native bladder wall in reconstructed bladders. Expression values were normalized to *Actg1* and *Hprt1*. Data are presented as mean ± SD, *p < 0.05, **p < 0.01, ***p < 0.001

Similarly, expression of endothelial cell markers—von Willebrand factor (vWf) and CD31—was significantly higher in bladders augmented with BAM seeded with ADSCs compared to unseeded BAM (*p* < 0.001). Increased expression of numerous angiogenesis markers including Vwf, CD31, and endoglin (Eng) was found in both: bladders reconstructed with BAM seeded with ADSCs and BAM only (Fig. 5a).

ADSCs did not influence regeneration of nerves and urothelium. Expression of both urothelial cell markers uroplakin 1b (Upk1b), occludin (Ocln), and zonula occludens protein 1 (Zo1), and neuronal cell markers neurofilament medium (Nefm), ubiquitin C-terminal hydrolase L1 (Uchl1), and adrenoceptor beta 3 (Adrb3) were significantly lower in reconstructed than in native bladder tissues (Fig. 4a, Fig. 5b).

Enhanced expression of fibroblast markers fibroblast activation protein alpha (Fap), S100 calcium-binding protein A4 (S100a4), and extracellular matrix protein fibronectin 1 (Fn1), was observed in bladders reconstructed with BAM only. Bladders reconstructed with BAM seeded with ADSCs displayed increased expression of S100a4 compared to native bladder tissues (*p* < 0.001)(Additional file 4: Figure S3AB).

### Immunofluorescence analysis of urothelium, smooth muscle, nerve, and vessel regeneration

**Fig. 6:**
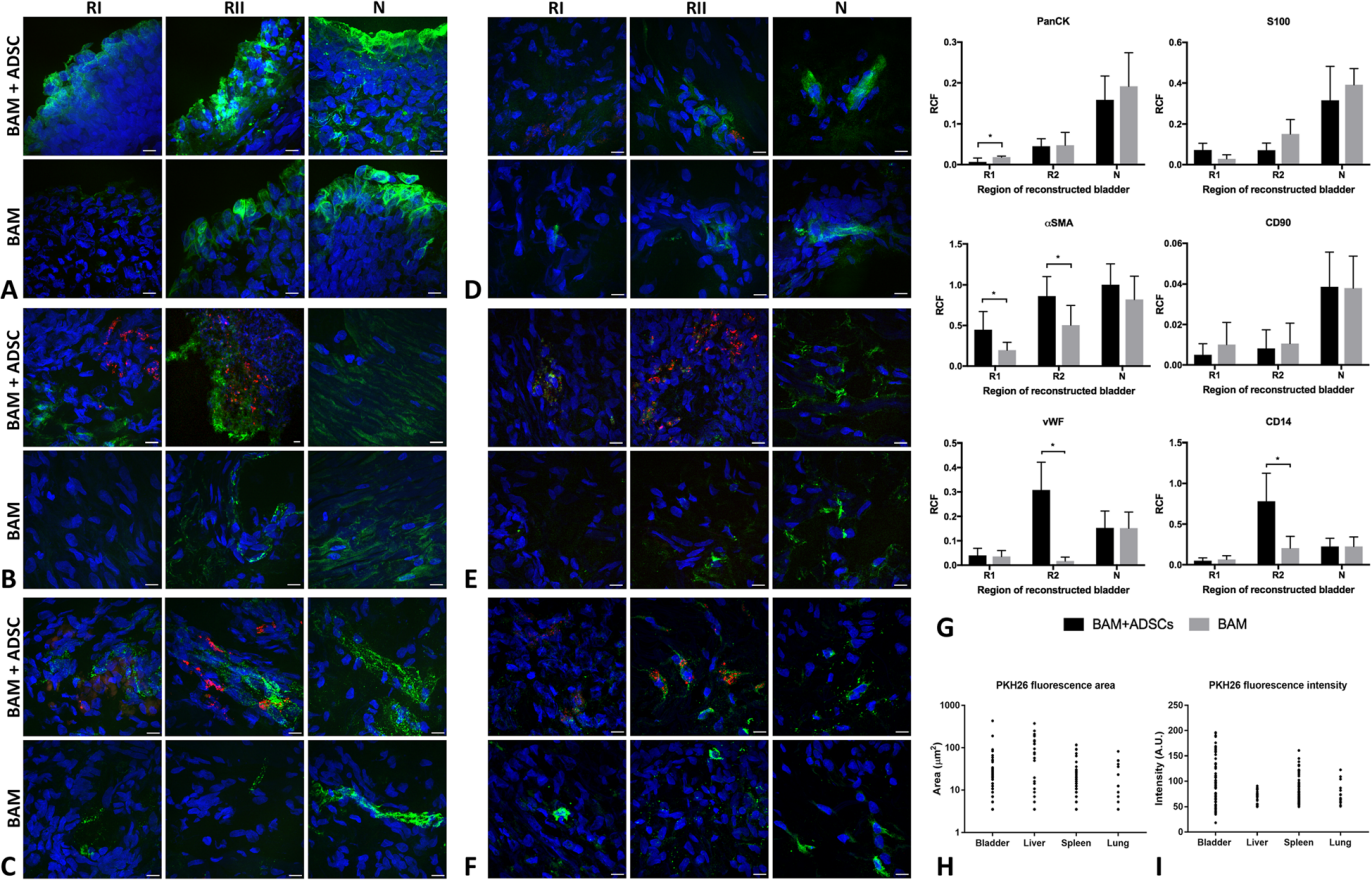
Immunofluorescence imaging of PanCK (**a**), αSMA (**b**), vWF (**c**), S100 (**d**), CD90 (**e**), CD14 (**f**) (green), PKH-26 (red), and DAPI (blue) in urinary bladders augmented with BAM seeded with ADSCs or BAM only. **g** Relative cell fluorescence (RCF) of PanCK, αSMA, vWF, S100, CD90, and CD14 in urinary bladders augmented with BAM seeded with ADSCs or BAM only. Data are presented as mean ± SD, *p < 0.05. R1—proximal graft region, R2—mid-graft region, N—native bladder wall in reconstructed bladders. **h**, **i** PKH-26-labeled cells tracking in urinary bladders and peripheral organs. Analysis of PKH-26 fluorescence area and intensity

Immunofluorescence analyses confirmed RT-PCR results. Enhanced angiogenesis and smooth muscle regeneration were observed in urinary bladders augmented with BAM seeded with ADSCs. Expression of αSMA was significantly higher in both regions (R1 and R2) of bladders reconstructed with BAM seeded with ADSCs compared to the bladders reconstructed with BAM only (R1 *p* < 0.05; R2 *p* < 0.01). However, comparable to native expression of αSMA was observed only in the mid-graft regions (R2) (*p* > 0.05) (Fig. 6b, g). Expression of vWF was significantly higher in the bladders reconstructed with BAM seeded with ADSCs compared to bladders reconstructed with BAM only (*p* < 0.05). The highest increase of vWF expression was observed in the mid-graft regions (R2) where the level of vWF was ~ 2 times higher compared to native bladder tissue (*p* < 0.05) (Fig. 6c, g). ADSCs did not influence expression of urothelial (PanCK), neuronal (S100), and mesenchymal stem cell (CD90) markers (Fig. 6a, d, e, g). Very low expression of PanCK and S100 indicated incomplete regeneration of urothelium and nerves in both graft center (R1) and mid-graft regions (R2) of reconstructed bladders (Fig. 6a, d, g). Expression of macrophage marker (CD14) was enhanced in the bladders reconstructed with BAM seeded with ADSCs compared to bladders reconstructed with BAM only (*p* < 0.05). The highest expression of CD14 with the level ~ 2 times higher compared to the native bladder wall (*p* < 0.05) was observed in the mid-graft region (R2) (Fig. 6f, g).

The colocalization of PKH26 with analyzed antigens was the highest for CD14 (*M =* 0.37), moderate for CD90 (*M =* 0.23), vWF (*M =* 0.21), and αSMA (*M =* 0.15), and the lowest for S100 (*M =* 0.01) and PanCK (*M =* 0). Tracking of PKH-26-labeled cells allows to find that only single ADSCs survived in the graft for 3 months follow-up (Fig. 6). PKH-26-labeled cells were also observed in peripheral organs including the lung, liver, and spleen (Fig. 6h, i). The number of PKH-26-labeled cells corresponding to PKH-26 fluorescence area and intensity was the highest in urinary bladder. Comparably high PKH-26 fluorescence area was detected in liver but their intensity was low which can indicate rather for the presence of PKH-26-labeled cellular debris than viable cells. On the other hand, proliferating cells expand their membrane size with each cell division and therefore may appear as weakly labeled. A significantly lower number of PKH-26-labeled cells was observed in the spleen and lung.

Analysis of PKH-26 colocalization showed that some of implanted ADSCs remained undifferentiated (*M =* 0.23 for CD90); others differentiated into endothelial (*M =* 0.21 for vWF) or smooth muscle cells (*M =* 0.15 for αSMA) and contributed to regeneration of vessels and muscular components of the bladder wall respectively. Most of implanted autologous ADSCs were phagocytosed by macrophages (*M =* 0.37 for CD14).

### Histological analysis of reconstructed bladders

In most cases, urothelium covered the inner surface of the reconstructed bladder wall with focally visible urothelial hyperplasia. In general, the urothelial layer was infiltrated by granulocytes migrating from thin-walled blood vessels. The regenerated smooth muscle layer characterized with irregular ultrastructure. Focally abundant fibrotic tissue overgrew regenerating smooth muscle. Inflammatory response was poorly pronounced which is limited to superficial layers of the reconstructed bladder wall. Quantitative analysis of regenerated bladder wall with differences between groups was demonstrated on Additional file 5: Figure S4.

## Discussion

The study revealed significantly better results of urinary bladder regeneration using BAM seeded with ADSCs than BAM alone; however, the quality of regenerated tissues was not homogenous. In general, the outcomes were tendentiously superior in the proximity to the native bladder wall. Regions adjacent to the intact wall characterized with well-developed vascular plexus and layered smooth muscle arrangement almost indistinguishable to normal one. In contrast, the central site of the graft was covered with starry scar. ADSCs not only reduced scaring but also stimulated smooth muscle regeneration and angiogenesis in the graft center. This observation is consistent with our understanding of endogenous regeneration which begins in the adjacent native bladder wall. The injury-activated urothelial, muscle, endothelial, and neural progenitor cells migrate toward the graft and spontaneously populate it.

It is hypothesized that stem cells trigger urinary bladder regeneration both directly by differentiation and indirectly by releasing trophic factors [8, 15]. At this point, we revealed that differentiation plays a minor role in the final regenerating effect mediated by ADSCs. Although implanted PKH-26-labeled ADSCs were reported to spontaneously differentiate into detrusor muscle αSMA-expressing cells, it was only a marginal outcome that could not be recognized as leading pathway of detrusor regeneration.

Microenvironment of the regenerating bladder wall is highly cytotoxic due to presence of urine which was demonstrated to kill predominantly undifferentiated stem cells. Even short 24 h ADSC incubation with urine reduced the colony viability by 90% [16]. Taking low chances for long ADSC survival might only provide temporary trophic boost for regenerating bladder tissue. In the present study, PKH-26-labeled cells were found 3 months after bladder reconstruction. PKH-26 signal was detected predominantly in regions where high expression of angiogenic and myogenic markers were identified. We came to the conclusion that ADSCs might form survival niches within graft including heterogonous cell population on different stages of mesenchymal differentiation.

Myogenic regeneration is known to be one of the major action fields for MSCs. We found that ADSCs enhanced smooth muscle regeneration in R2 of the reconstructed bladder. PKH-26-labeled cells represented only a small fraction of αSMA-expressing cells within regenerating tissue. These results demonstrated that cellular ingrowth from surrounding tissue contributes more significantly to developing smooth muscles than implanted stem cells. In contrast, Sharma et al. reported that traced MSCs predominantly underwent myogenic differentiation in tissue-engineered bladders [9]. Ability to support detrusor regrowth constituted MSCs as a major cell type evaluated for induced bladder regeneration [12].

Angiogenesis markers were elevated in the R2 region of the reconstructed bladder close to the native bladder wall. Only CD105 expression was increased in the graft’s center (R1) probably because of known CD105 involvement in TGF-ß pathway [17]. The highest TGF-ß expression was to be expected in the most fibrous region. Interesting is overlapping elevated expression of vWF with CD14 macrophages in the mid-graft region in bladders augmented with ADSC-seeded BAM. CD14 cells described as monocyte/macrophage linage are recruited to build new vascular plexus of regenerating tissue [18]. The colocalization of PKH-26 with both CD14 and vWF indicated that implanted ADSCs were involved in the angiogenesis process. Alternatively, regions with overlapping PKH-26 and CD14-positive signals might indicate areas where ADSCs were phagocytosed by infiltrating macrophages.

The pattern of urothelium markers identified in the reconstructed bladder wall exposed significant divergence with a proper one. Disturbing is the decreased level of uroplakin family because these proteins regulate membrane permeability of superficial umbrella cells. If urothelial cells fail to restore the impermeable layer, the urine penetrates deep into the graft eliminating cells rebuilding the neo-bladder wall. Most of available studies reported as a success the identification of a few urothelial markers within the superficial layer of tissue-engineered bladder wall [19, 20]. We must however admit based on analysis of 10 cardinal urothelium markers that regenerated urothelial layer differed in many aspects from a normal one. Moreover, in comparison to presented critical results of molecular analysis, the standard evaluation based on H&E staining might create an illusion of well-underwent urothelium regeneration.

The neural regeneration demands a favorable environment to take place; hence, severed axons fail to penetrate beyond the lesion site [21]. Paracrine stimulation delivered by MSCs alone seemed to be insufficient to initiate neuronal regrowth.

PKH-26-labeled cells in 3 months follow-up were observed not only in the reconstructed bladders but also in peripheral organs including the lung, liver, and spleen. Successful homing of systematically administrated MSCs to bone marrow, liver, or lungs was documented. The mechanisms governing MSC migration were not however fully elucidated [22]. Nevertheless, these regulation patterns are corresponding to those existing during embryogenesis when stem cells invade forming tissues and organs. Migration capacity is a cardinal feature of adult MSCs [23]. In this situation, seeded ADSCs on BAM biomaterial scaffold might behave similarly to metastatic cells after epithelial to mesenchymal transition. Therefore, ADSCs can migrate within the recipient’s tissues and move through the lymphatic and circulatory systems. Analogue mechanism to “leucocyte homing” involving multistage transmigration through the endothelium and the underlying basement membrane is responsible for MSC’s mobility [24].

An alternative explanation of PKH-26 cell presence in the lungs, spleen, and liver might be related to macrophage phagocytosis. Detected PKH-26 signal could come from ADSC debris phagocytosed by macrophages.

Our study showed that currently used tissue engineering techniques for urinary bladder regeneration do not allow for translation of this technology to standard clinical practice. ADSCs support regeneration of large defects of the urinary bladder wall but the process is incomplete in the central graft region. Only a marginal percentage of implanted stem cells survive and differentiate into smooth muscle and endothelial cells. The main obstacles on the road to successful urinary bladder regeneration are cytotoxic influence of urine on implanted cells and insufficient graft revascularization which lead to cell death and graft fibrosis. One of the most promising approaches to resolve these problems would be construction of an appropriate scaffold providing a vascular network and protecting implanted cells against toxic influence of urine by bio-printing technology. Ideally, a vascular network should be developed and intercorporate with the host vascular system by adequate anastomosis. Unfortunately, this approach is currently far beyond tissue engineering technology. There are described experimental methods dedicated to generate artificial vascular networks but they are not efficient and reliable enough to be used in clinics. To date, the best option is to improve natural neo-angiogenesis by creating biomaterials guiding sprouting vessels or transplanting cells enhancing this process, e.g., endothelial progenitor cells.

## Additional files

Additional file 1: **Figure S1.** Overview of the experimental workflow. (A) Isolation of ADSCs from subcutaneous adipose tissue. (B) ADSCs in vitro cultivation. (C) Urinary bladder stepwise decellularization and generation of BAM scaffold. (D) ADSCs labelling with PKH-26 fluorescent dye and seeding on BAM scaffold. (E) Preparation of acellular and autologous cellular graft for bladder wall substitution. (F) Urinary bladder augmentation after hemicystectomy. (G) Harvesting of tissue-engineered urinary bladder wall after 3 months follow-up. (H) Evaluation of PKH-26 colocalization with expression of urothelial, myogenic and neuronal markers. (TIF 1257 kb)

Additional file 2: **Table S1.** Primer Sequences for Quantitative Real-Time PCR. (DOC 97 kb)

Additional file 3: **Figure S2.** (A) Immunophenotypic characterization of ADSCs. Fluorescein isothiocyanate (FITC), and phycoerythrin (PE) conjugated antibodies were used for phenotyping of the ADSCs by flow cytometry. Gray peaks represents isotype staining; and red peaks, antigen-specific staining. Flow cytometry confirmed the ADSCs immunophenotype with high expression of CD29, CD44 and CD90 surface markers and low expression of CD11b, CD31 and CD45 surface markers. (B) Differentiation potential of ADSCs: a positive Oil Red O staining of lipid vacuoles after adipogenic induction (bar 200 μm); Alcian Blue staining of proteoglycans after chondrogenic induction (bar 100 μm), Alizarin Red staining of mineral deposits after osteogenic induction (bar 200 μm). (C) ADSCs cultured in a standard medium remained undifferentiated. (TIF 7581 kb)

Additional file 4: **Figure S3.** Expression of fibroblast (A) and extracellular matrix protein (B) markers in urinary bladders augmented with BAM seeded with ADSCs or BAM only determined by RT-PCR. R1—proximal graft region, R2—mid-graft region, N—native bladder wall in reconstructed bladders. Expression values were normalized to *Actg1* and *Hprt1*. Data are presented as mean ± SD, *p < 0.05, **p < 0.01, ***p < 0.001. (TIF 625 kb)

Additional file 5: **Figure S4.** Morphological analysis of urothelial, smooth muscle and serosal layers and inflammatory reaction in bladders augmented with BAM seeded with ADSCs and BAM only. Urothelium was assessed as 3 normal, 2 segmental, 1 focal, 0 absent. Smooth muscle was assessed as 3 normal, 2 normal, segmentally disrupted with regrowth regions, mixed with fibrotic tissue, 1 in total regrowth, mixed with fibrotic tissue, 0 absent. Serosa was assessed as 3 normal, 2 normal with granulocytic infiltrates, 1 segmental, 0 absent. The intensity of inflammatory infiltration was assessed as 3 severe, 2 moderate, 1 minimal, 0 lack. (TIF 1161 kb)

## Abbreviations

Adrb3: Adrenoceptor Beta 3
ADSCs: Adipose-derived stem cells
BAM: Bladder acellular matrix
Cald1: Caldesmon 1
Cnn: Calponin
Des: Desmin
Eng: Endoglin
Fap: Fibroblast activation protein alpha
Fn1: Fibronectin 1
Myh11: Myosin heavy chain 11
Mylk: Myosin light chain kinase
Nefm: Neurofilament medium
Ocln: Occludin
panCK: Pan cytokeratin
S100a4: S100 calcium-binding protein A4
Smtn: Smoothelin
Tagln: Transgelin
Uchl1: Ubiquitin C-terminal hydrolase L1
Upk1b: Uroplakin 1b
Vcl: Vinculin
vWF: von Willebrand Factor
Zo1: Zonula occludens protein 1
αSMA: Alpha smooth muscle actin
