# Supplementary figures and images for: Understanding the role of mesenchymal stem cells in urinary bladder regeneration—a preclinical study on a porcine model
Source: Stem Cell Res Ther. 2018 Nov 28;9:328. doi: 10.1186/s13287-018-1070-3 (PMC6260700; doi:10.1186/s13287-018-1070-3)

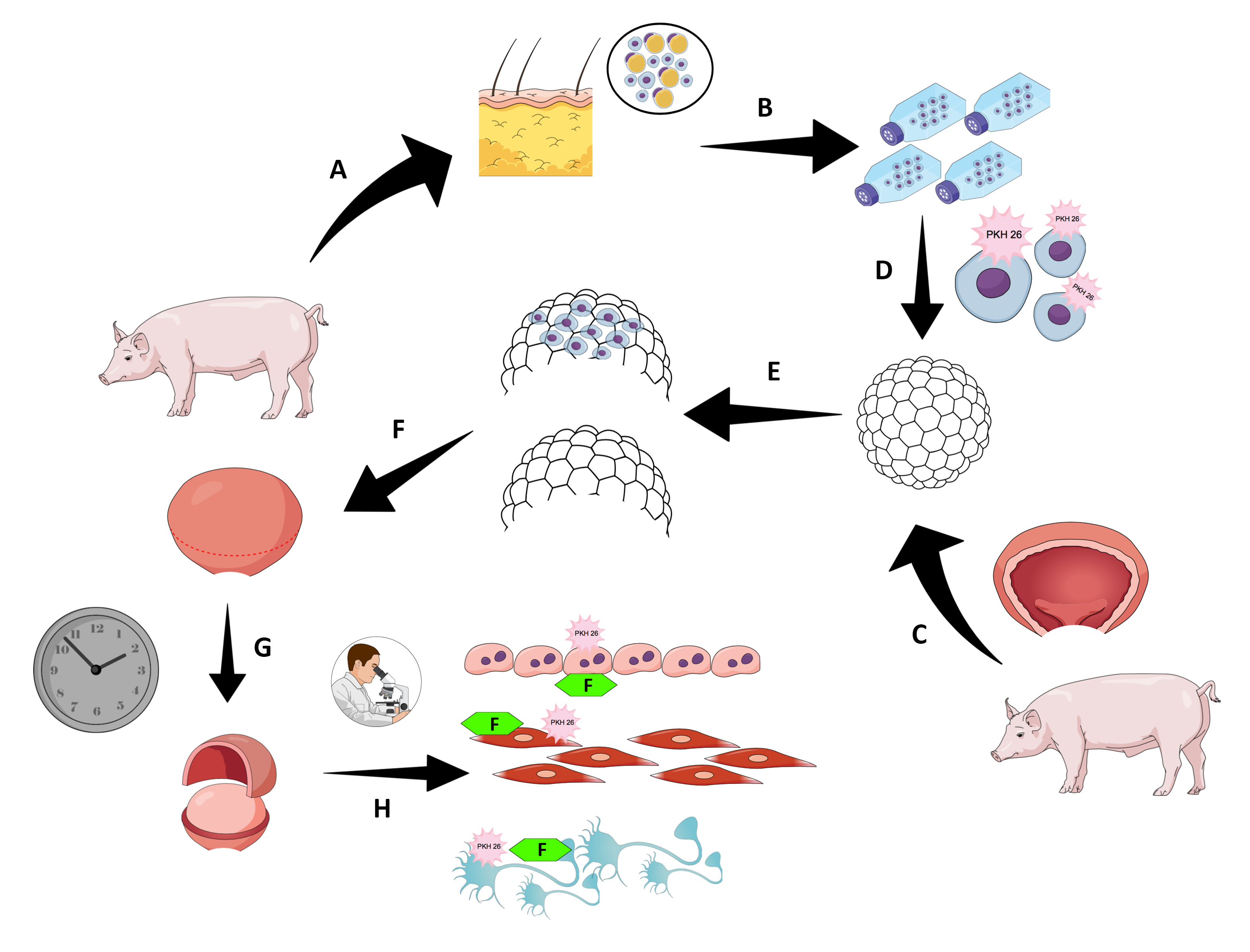

Supplement: Supplementary file 1 — Figure S1. Overview of the experimental workflow. (A) Isolation of ADSCs from subcutaneous adipose tissue. (B) ADSCs in vitro cultivation. (C) Urinary bladder stepwise decellularization and generation of BAM scaffold. (D) ADSCs labelling with PKH-26 fluorescent dye and seeding on BAM scaffold. (E) Preparation of acellular and autologous cellular graft for bladder wall substitution. (F) Urinary bladder augmentation after hemicystectomy. (G) Harvesting of tissue-engineered urinary bladder wall after 3 months follow-up. (H) Evaluation of PKH-26 colocalization with expression of urothelial, myogenic and neuronal markers. (TIF 1257 kb) [file 13287_2018_1070_MOESM1_ESM.tif]

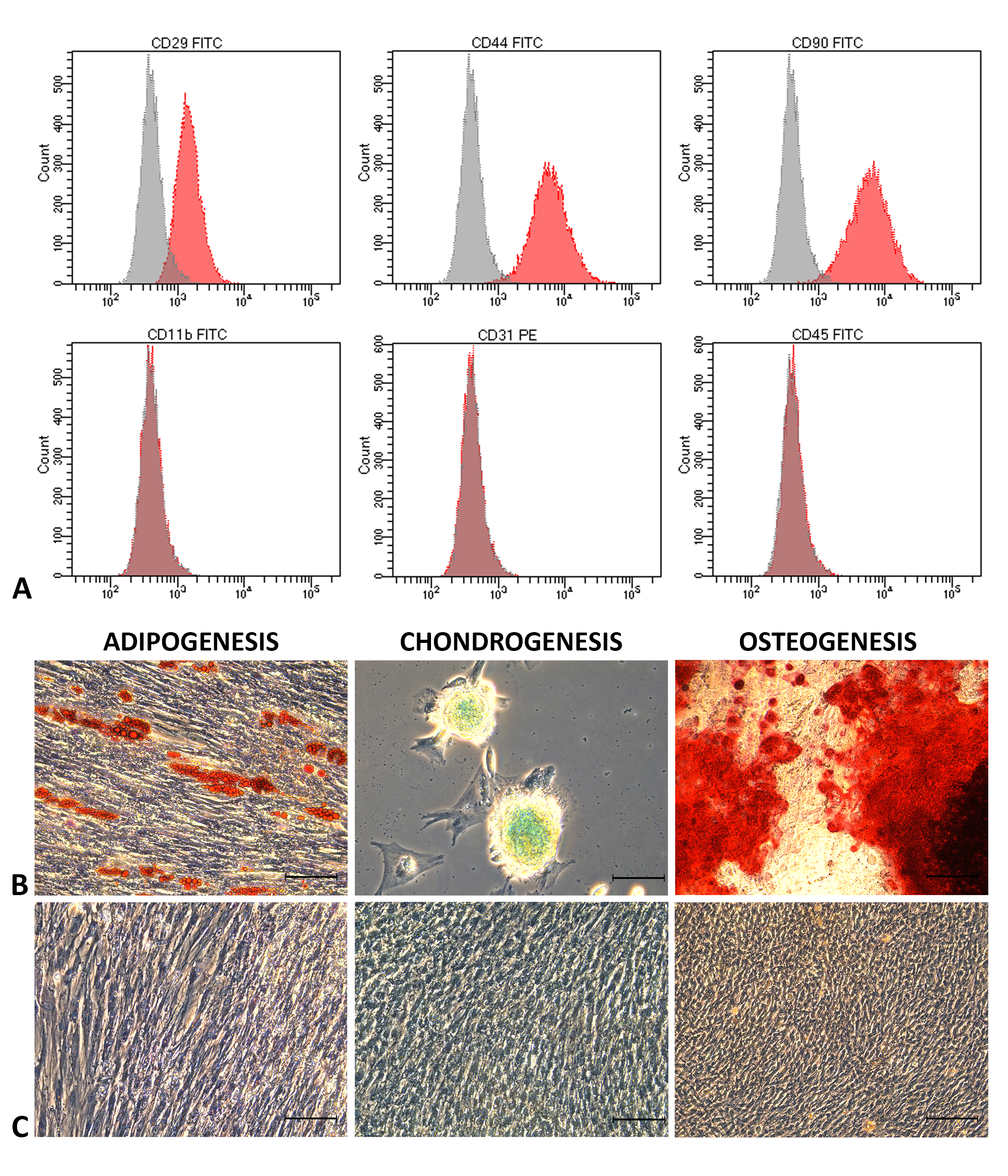

Supplement: Supplementary file 3 — Figure S2. (A) Immunophenotypic characterization of ADSCs. Fluorescein isothiocyanate (FITC), and phycoerythrin (PE) conjugated antibodies were used for phenotyping of the ADSCs by flow cytometry. Gray peaks represents isotype staining; and red peaks, antigen-specific staining. Flow cytometry confirmed the ADSCs immunophenotype with high expression of CD29, CD44 and CD90 surface markers and low expression of CD11b, CD31 and CD45 surface markers. (B) Differentiation potential of ADSCs: a positive Oil Red O staining of lipid vacuoles after adipogenic induction (bar 200 μm); Alcian Blue staining of proteoglycans after chondrogenic induction (bar 100 μm), Alizarin Red staining of mineral deposits after osteogenic induction (bar 200 μm). (C) ADSCs cultured in a standard medium remained undifferentiated. (TIF 7581 kb) [file 13287_2018_1070_MOESM3_ESM.tif]

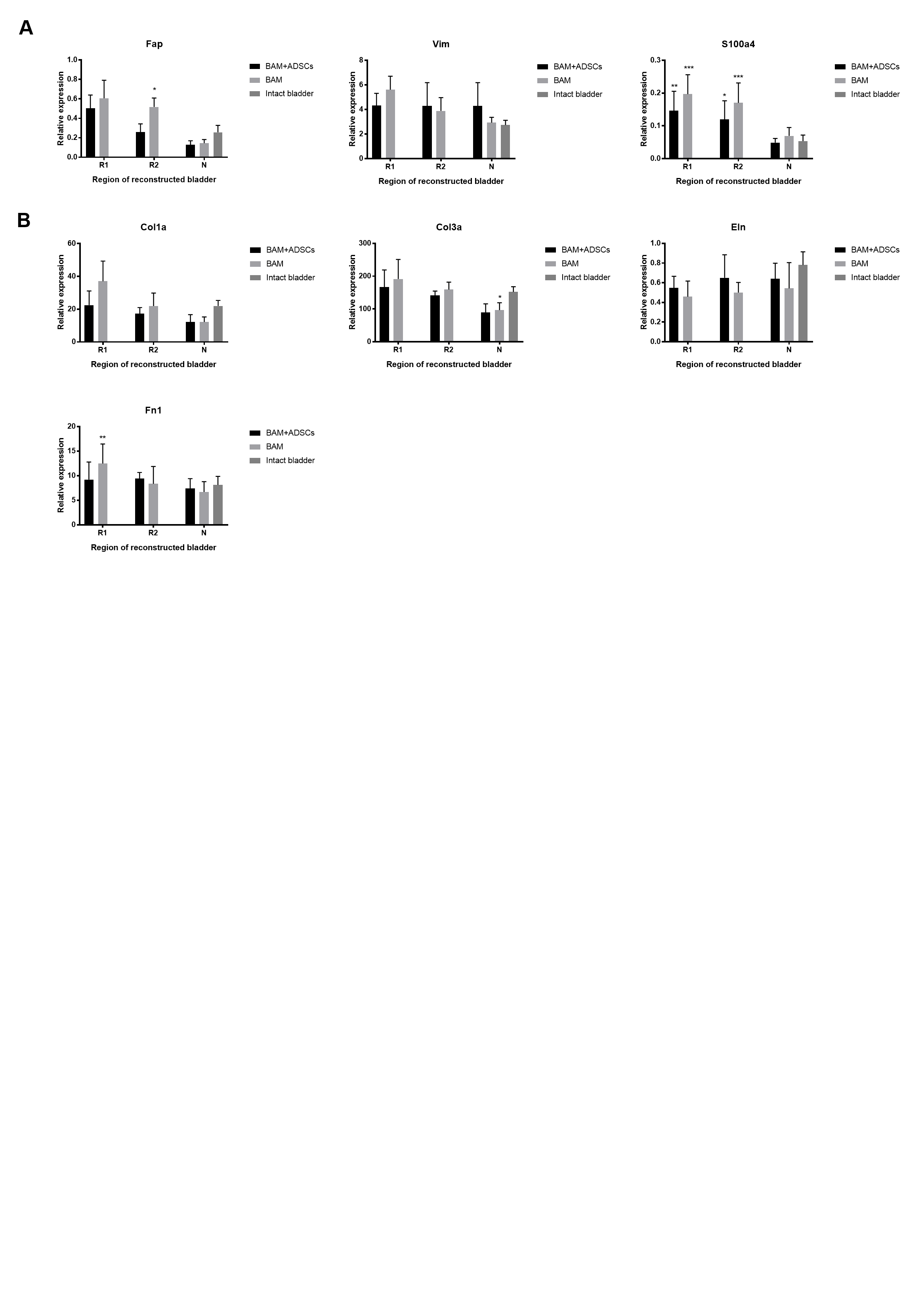

Supplement: Supplementary file 4 — Figure S3. Expression of fibroblast (A) and extracellular matrix protein (B) markers in urinary bladders augmented with BAM seeded with ADSCs or BAM only determined by RT-PCR. R1—proximal graft region, R2—mid-graft region, N—native bladder wall in reconstructed bladders. Expression values were normalized to Actg1 and Hprt1. Data are presented as mean ± SD, *p < 0.05, **p < 0.01, ***p < 0.001. (TIF 625 kb) [file 13287_2018_1070_MOESM4_ESM.tif]

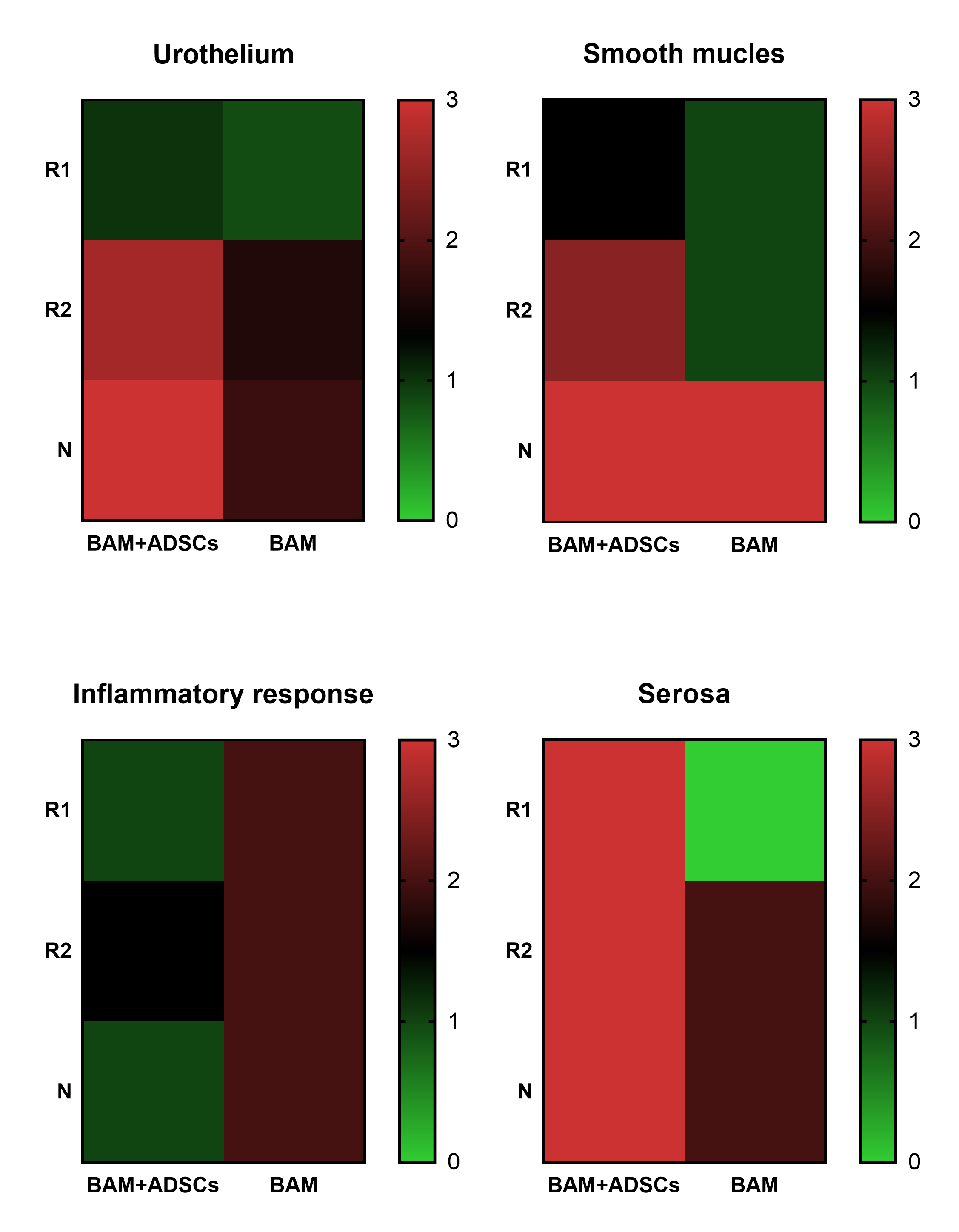

Supplement: Supplementary file 5 — Figure S4. Morphological analysis of urothelial, smooth muscle and serosal layers and inflammatory reaction in bladders augmented with BAM seeded with ADSCs and BAM only. Urothelium was assessed as 3 normal, 2 segmental, 1 focal, 0 absent. Smooth muscle was assessed as 3 normal, 2 normal, segmentally disrupted with regrowth regions, mixed with fibrotic tissue, 1 in total regrowth, mixed with fibrotic tissue, 0 absent. Serosa was assessed as 3 normal, 2 normal with granulocytic infiltrates, 1 segmental, 0 absent. The intensity of inflammatory infiltration was assessed as 3 severe, 2 moderate, 1 minimal, 0 lack. (TIF 1161 kb) [file 13287_2018_1070_MOESM5_ESM.tif]
