# Supplementary material for: Understanding the role of mesenchymal stem cells in urinary bladder regeneration—a preclinical study on a porcine model
Source: Stem Cell Res Ther. 2018 Nov 28;9:328. doi: 10.1186/s13287-018-1070-3 (PMC6260700; doi:10.1186/s13287-018-1070-3)
Supplement: Supplementary file 2 — Table S1. Primer Sequences for Quantitative Real-Time PCR. (DOC 97 kb) [file 13287_2018_1070_MOESM2_ESM.doc]

**Table S1: Primer Sequences for Quantitative Real-Time PCR.**

|  |  |  | |  |
| --- | --- | --- | --- | --- |
| **Gene Symbol** | **GenBank** | **5' Primer** | | **3' Primer** |
| ACTA2 | NM_001164650 | | AATACTCCGTCTGGATTGGTG | GGCTTCGTCGTACTCCTGTTTG |
| ACTG1 | XM_003357928 | | GCTACAGCTTCACCACCAC | GCCATCTCCTGCTCGAAGTC |
| ADRB3 | NM_001099927 | | TTGCCCTTCTTTGTGGTCAAC | AAGTCTGGGCTGTGGCAGTAG |
| B2M | NM_213978 | | TTCAGCAAGGACTGGTCTTTC | TATACTGATCCACAGCGTTAGGAG |
| CALD1 | XM_003134653 | | AAGAAAGGAAAGCCACCAAG | AGTCAATGCTAACTCAGGCACTG |
| CDH5 | NM_001001649 | | GCACCGACTCATCCGACTC | CTAATACAGCAGCTCCTCTCG |
| CNN1 | NM_213878 | | AGCAGGAGCTTCGAGAGTG | GCCGTCCATGAAGTTGTTG |
| COL1A1 | XM_013981006 | | GGCAAGAACGGAGATGATG | CACCATCCAAACCACTGAAAC |
| COL3A1 | NM_001243297 | | TATCGAACACGCAAGGCTG | CCAATGTCCGCACCAAATTC |
| DES | NM_001001535 | | GCACTAACGATTCCCTGATGAG | GTGCGATGTTGTCCTGGTAG |
| ELN | XM_013995554 | | ACCCTTTGGAGGTCAGCAG | CAGCTTGGGTGCCTTGATG |
| ENG | NM_214031 | | CATCACGAGCCTGACCTTC | GAGTAGGTGCCACGCAAGAC |
| FAP | XM_005671884 | | GGTACTCAGACCAGAACCACG | GGCAGAGAGAAACATTGCTTTAG |
| FLT1 | XM_001925740 | | GAGAGGAAGATTTCGTGCTGC | TTAGACGGGAGGGCTGGAG |
| FN1 | XM_003133641 | | AAGCGACGTGTTATGATGATG | AGGAGCAAATGGCACCGAG |
| GAPDH | NM_001206359 | | TCAGCAATGCCTCCTGCAC | CATGAGTCCCTCCACGATG |
| HPRT1 | NM_001032376 | | TTGGGTGGATTGTTGTTTGAC | TCATTCAATAGTGGTGTGGTTTAAG |
| KDR | XM_003128987 | | TCTGGGTATCACTCGGACGAC | AGGATCTGAGCTGCACTGC |
| KRT18 | XM_005652579 | | GCTGCACCTGGAGTCAGAG | TCCAGCTTGACCTTGATGTTC |
| KRT19 | XM_003131437 | | GGTCAGTGTGGAGGTTGATTC | TCATACTGGCTTCTCATGTCG |
| KRT20 | XM_003131462 | | GGTGAGCTATGGGAGCGAC | GCGGTCATTGAGGTTCTTC |
| KRT8 | NM_001159615 | | AGGCGACTGCTTAGGCTTC | AGACATTTGAATTGGCTTGGAG |
| MCAM | XM_003129935 | | TGGAGGAAGAAAGTACCGATG | CTCAGCAGCGATACTGTGGTC |
| MYH11 | XM_013991919 | | TGAACTCCGCTTAGCACTGTC | AACTTAAATTGCTGCCCTGAAC |
| MYLK | XM_005670159 | | ATTGACGAGGACTTTGAGCTG | GGTGCCCGTCTTGTTGACAC |
| NEFM | XM_005670439 | | TCCTCAGTCTTTGGGTGATTC | TGGCTCAGTTGGTTCTTCG |
| OCLN | NM_001163647 | | AGGCCAAACCTTTCTGGTC | TCCTTCCTTGGAGTGTTCAGAC |
| PECAM1 | NM_213907 | | CCTGGAAGAAAGGACTCATCG | CCAAGTAACAAGACGGCAATG |
| S100A4 | NM_001252605 | | GCTGATGAGCAACCTGGACAG | GAAGACGCAGTACTCCTGGAAG |
| SELE | NM_214268 | | AATGGGACTTGCTGCTGGTG | TCCGAAGGCGTTTCAGGAG |
| SMTN | NM_001244360 | | CATCTGATTCTCCTATGGCTGC | GTTGAGGGACCGAGGGTTC |
| SYP | XM_003135078 | | GGAACTACTCCTCATCGGCTG | CCTTTGTTGTTCTCTCGGTACTTG |
| TAGLN | NM_001244150 | | TGGTTTATGAAGAAAGCCCAG | ACTGATGATCTGCCGAGGTC |
| UCHL1 | NM_213763 | | TATGAACTTGATGGTCGGATG | CAGCAGCGAGTCCTCTGAG |
| UPK1A | NM_001123211 | | CCTGGTGCTCATGCTCATC | TCTCGGTGGGTATAGGACG |
| UPK1B | NM_001123212 | | CAATATAGCCTCTACCCGCTG | GCCGACAAATATGCCAATC |
| UPK2 | NM_214012 | | AGGGAGCTGGTGAGTGTAGTG | TAGTATTTGGTTCCTGGCACAAG |
| UPK3A | XM_003125969 | | GCATGTTTGACAGCTCAGC | CGACCAGGACATAGAGATAGACCTC |
| VCAM1 | NM_213891 | | AAGCAGAGACAGGCGACAC | TTCATTCTTGGATTCACATTCG |
| VCL | NM_213934 | | AAGATGATTGACGAGAGGCAG | CGGAAATGAGAACTGGCAAC |
| VIM | XM_005668106 | | GCCGTGGAAGCTGCTAACTAC | TAGGTGGCAATCTCAATGTCG |
| VWF | NM_001246221 | | GCAGTACCTCTCGGAGCACAG | GGCATCCGCTTGATCTCATC |
| ZO-1 | XM_013993251 | | TCCAGAGGGAGCATCTAAGC | GCAGTGTTCTGTCATGTTCG |
|  |  |  | |  |
